# Thorough consideration of electroconvulsive therapy (ECT) in treatment-resistant psychiatric disorders

**DOI:** 10.1038/s41380-022-01665-w

**Published:** 2022-06-22

**Authors:** David Zilles-Wegner, Charles H. Kellner, Alexander Sartorius

**Affiliations:** 1 University Medical Center Göttingen, Department of Psychiatry and Psychotherapy, von-Siebold-Str. 5, D-37075 Göttingen, Germany; 2 Department of Psychiatry and Behavioral Sciences, Medical University of South Carolina, Charleston, SC USA; 3 Department of Psychiatry and Psychotherapy, Central Institute of Mental Health (CIMH), Medical Faculty Mannheim, University of Heidelberg, Square J 5, D-68159 Mannheim, Germany

**Keywords:** Depression, Schizophrenia

## To the Editor:

The article by Howes et al. [1] presents a valuable framework and broad access to the topic of treatment-resistant disorders in psychiatry and deserves to be read by both researchers and clinicians. Nevertheless, we would like to comment on the too short and maybe even misleading passage on electroconvulsive therapy (ECT).

The relative importance of ECT for the treatment of the most severe and treatment-resistant disorders in psychiatry and its potential for a better understanding of the neurobiology of treatment response [2, 3] should merit more than only three sentences in an otherwise comprehensive overview. From a practitioner’s viewpoint, ECT is much more than an approved therapy for “treatment-resistant depression and mania” [4]. There is further clear evidence for its effectiveness in schizophrenia [5] and catatonia [6], and ECT is recognized as a standard treatment in guidelines from major psychiatric associations [4].

Beyond this clinical perspective, the authors note that ECT’s mechanism of action is only poorly defined. Not to forget: If we perfectly understood how ECT works, we would as a corollary have perfectly understood the pathophysiology of some of the most impairing psychiatric disorders. Instead of citing recent reviews on mechanisms of action of ECT [7] the authors (hopefully inadvertently) cite a review article from one of the most obvious ideological opponents of ECT, who concludes the abstract of his article by saying that “ECT affects the brain in a similar manner as severe stress or brain trauma”, which obviously is not true [8]. On the contrary, there are several replicated findings that may well represent physiologically relevant factors that are in part associated with, and contribute to, clinical response [9]. Meta-analytic evidence suggests regionally specific brain volume increases, e.g. in the hippocampal region [10]. Moreover, there is corroborative evidence for increased levels of neurotrophic factors after ECT [11] and the reduction of systemic inflammation in the treatment course [12], all factors that correspond well to current hypotheses on depression pathophysiology [13].

To conclude, we think that ECT should be given the consideration it deserves—from both a clinical and scientific perspective—to exploit its full potential for the treatment of severely affected patients, identification of biological markers of treatment response, and the elucidation of the pathophysiology of treatment-resistant psychiatric disorders. For patients with the most serious forms of treatment-resistant mood and psychotic disorders, ECT should be a standard, go-to treatment, not an afterthought.
